# Efficacy of Artesunate + Sulfamethoxypyrazine/Pyrimethamine versus Praziquantel in the Treatment of *Schistosoma haematobium* in Children

**DOI:** 10.1371/journal.pone.0006732

**Published:** 2009-10-05

**Authors:** Mahamadou S. Sissoko, Abdoulaye Dabo, Hamidou Traoré, Mouctar Diallo, Boubacar Traoré, Drissa Konaté, Boubacar Niaré, Moussa Diakité, Bourama Kamaté, Abdrahamane Traoré, Aboudramane Bathily, Amadou Tapily, Ousmane B. Touré, Sarah Cauwenbergh, Herwig F. Jansen, Ogobara K. Doumbo

**Affiliations:** 1 Department of Epidemiology of Parasitic Diseases, Faculty of Medicine, Pharmacy and Odonto-Stomatology, Malaria Research and Training Center, University of Bamako, Bamako, Mali; 2 Dafra Pharma nv, Turnhout, Belgium; Swiss Tropical Institute, Switzerland

## Abstract

**Background:**

This study was conducted to determine the efficacy of the antimalarial artemisinin-based combination therapy (ACT) artesunate +sulfamethoxypyrazine/pyrimethamine (As+SMP), administered in doses used for malaria, to treat *Schistosoma haematobium* in school aged children.

**Methodology/Principal Findings:**

The study was conducted in Djalakorodji, a peri-urban area of Bamako, Mali, using a double blind setup in which As+SMP was compared with praziquantel (PZQ). Urine samples were examined for *Schistosoma haematobium* on days −1, 0, 28 and 29. Detection of haematuria, and haematological and biochemical exams were conducted on day 0 and day 28. Clinical exams were performed on days 0, 1, 2, and 28. A total of 800 children were included in the trial. The cure rate obtained without viability testing was 43.9% in the As+SMP group versus 53% in the PZQ group (Chi^2^ = 6.44, p = 0.011). Egg reduction rates were 95.6% with PZQ in comparison with 92.8% with As+SMP, p = 0.096. The proportion of participants who experienced adverse events related to the medication was 0.5% (2/400) in As+SMP treated children compared to 2.3% (9/399) in the PZQ group (p = 0.033). Abdominal pain and vomiting were the most frequent adverse events in both treatment arms. All adverse events were categorized as mild.

**Conclusions/Significance:**

The study demonstrates that PZQ was more effective than As+SMP for treating *Schistosoma haematobium*. However, the safety and tolerability profile of As+SMP was similar to that seen with PZQ. Our findings suggest that further investigations seem justifiable to determine the dose/efficacy/safety pattern of As+SMP in the treatment of *Schistosoma* infections.

**Trial Registration:**

ClinicalTrials.gov NCT00510159

## Introduction

An estimated 200 million individuals worldwide are living with schistosomiasis, the second parasitic disease after malaria in terms of socioeconomic and public health importance [**1]**, [2****]. From the estimated global burden of 50 million DALYs (disability adjusted life years) for the cluster of neglected diseases, schistosomiasis accounts for 1,760,000 DALYs [**3**]. Since no vaccine is available at this time, reduction of morbidity has become the most important goal for all schistosomiasis control programs. Currently, the standard first line treatment is praziquantel (PZQ). This drug is easy to administer and is active against all *Schistosoma* species. However, low cure rates have been observed in several regions throughout Africa after praziquantel treatment [**4]**, [5****]. In highly endemic areas, treatment with the standard dose of 40 mg/kg may no longer suffice. As a consequence, higher doses may be given over a longer period of time, which may lead to the appearance of more side effects, which in turn may have a major impact on treatment compliance, as well as an increased risk of resistance. Besides, praziquantel is only active against the adult forms of the parasite, and does not clear young developing forms in the patient. Therefore, egg production will eventually resume, and infection continues to spread, making control efforts difficult and of limited efficacy.

Artemether and artesunate, derivates of artemisinin (the Chinese drug *qinghaosu*) are widely and effectively used against malaria, particularly as artemisinin-based combination therapy (ACT) [**6**]. Co-infection with schistosomes is common in malaria endemic areas. For example, in Mali, 76.6% of *Schistosoma haematobium* infected children develop malaria [**7**]. The artemisinins have interesting antischistosomal properties, in particular against the juvenile, 2- to 3- week – old, parasites [**8**]. From the limited number of studies (*in vivo* & *in vitro*) conducted on *Schistosoma spp* with artemisinin derivatives, higher cure rates were obtained when artesunate was used in combination with praziquantel in comparison to cure rates of artemether or artesunate alone [**9]**, [10], [11****]. Although several publications from pilot studies in humans demonstrate the effects of artemisinin derivatives used alone [**12]**, [13****], pilot studies that were conducted with ACTs seem to demonstrate even higher efficacy [**11]**, [14], [15****]. These findings suggest an interesting synergy, although more elaborate and statistically powerful trials are necessary to elucidate these findings [**16**].

In this study, the safety and effect of the ACT Co-Arinate FDC® (combination of artesunate with sulfamethoxypyrazine and pyrimethamine (As+SMP)) and praziquantel (PZQ) on the cure rate and egg reduction rate of *Schistosoma haematobium*, the most widely distributed species in Sub-Saharan African countries, were compared in a double blind controlled trial.

## Materials and Methods

The protocol for this trial and supporting CONSORT checklist are available as supporting information; see Checklist S1 and Protocol S1.

### Study site

The trial was carried out between August 2007 and October 2007 in Djalakorodji, a peri-urban area of Bamako, the capital of Mali. The village is situated to the north of Bamako and has 28,000 inhabitants. Numerous temporary streams, created by seasonal rains, intersect the village in several locations. Although these streams dry up after the rainy season (March - April) they are used by the population for multiple activities such as laundry, dishwashing, fishing, bathing and recreation. They constitute an excellent breeding place for snails, the intermediate hosts necessary for completion of the *Schistosoma* life cycle. Consequently, repetitive *Schistosoma* infections are common, particularly in children. Risk of contamination is especially high during the period following the rainy season until these temporary water sources have dried up. These excellent conditions for *Schistosoma* proliferation lead to incidences of up to 70% in schoolchildren [**17**].

### Study design

This is a double-blind, controlled clinical trial in children, aged 6 to 15 years. The study was designed to determine the efficacy of the antimalarial ACT As+SMP, administered in doses used for malaria, to treat *S. haematobium* and to determine the safety profile during treatment and follow up. The trial was approved by the Institutional Review Board of the Faculty of Medicine, Pharmacy and Dentistry, University of Bamako, Mali. The study objectives were explained in detail to the authorities of the village. After receiving approval for conducting the study in the community, all the children's parents or guardians were invited to enrol their children into the study at the nearest schools according to a schedule broadcasted on the local radio as well as by the local informer (‘Le Crieur Public’). During the meeting, detailed information was provided about the goals, procedures and potential risks of the study. After obtaining individual written informed consent, all children were invited to bring urine samples in a plastic container for examination. The following day, those children who were excreting *S. haematobium* eggs were eligible for enrolment into the study. To confirm their positive diagnosis for schistosomiasis, the enrolled children were asked to provide another urine sample for examination the following day and the mean egg count of the two samples was considered as the baseline intensity of infection. At the beginning of the trial, the study physician carried out a full clinical examination of all schistosome positive children, including assessment of liver and spleen enlargement and anaemia. The children included into the study were between 6 and 15 years old, appeared healthy at enrolment as assessed by the study physician and suffered from *S. haematobium* infection (excreting eggs in urine). They were all residents of Djalakorodji, were able to receive oral treatment, suffered of no apparent chronic or debilitating conditions and the respective parent/legal guardian gave written informed consent to participate in the study, after they had been provided with detailed study information by the investigators. The following exclusion criteria applied: weighing more than 50 kg, pregnant or lactating at the time of the study, presence of severe illness such as cerebral cysticercosis or signs of severe malnutrition (defined as children with weight/height ratio below 3 standard deviations or below 70% of the median of the Word Health Organization's (WHO) standardized reference values, or still with symmetrical oedema affecting both feet). Children were also excluded if they were hypersensitive to As, SMP or PZQ, used another ACT or anti-schistosomal drug during the study, or previously participated in another similar study.

The cure rate for the PZQ group was assumed to be 75% (60–95%), while the cure rate in the As+SMP treatment group was estimated at +10% in comparison to the PZQ group. With 90% power and a two-sided type 1 (α) error of 5%, we calculated that 354 patients were needed in each of the treatment arms. 46 additional children were included in each arm to correct for loss to follow-up and non compliance. A total number of 800 children were enrolled in the trial, with 400 children randomized into each arm. Block randomization was used to allocate the patients to the two treatment arms, and was blinded to the treating physician and his team, as well as to the patient.

One arm of the study received PZQ (treatment B) on the first day of treatment and As+SMP matched placebo (treatment D) on the second day of treatment. The second arm received PZQ matched placebo (treatment A) on the first day of treatment and As+SMP (treatment C) on the second day of treatment.

Treatment allocation was carried out using an envelope system, containing either “treatment A/C” or “treatment B/D”. All tablets were administered under direct medical supervision, which assures compliance to the treatment. The random allocation sequence was computer generated in Belgium. The participants were enrolled by the clinical investigators and they were assigned to their treatment group by the study pharmacist on site (Djalakorodji, Mali).

Co-Arinate FDC® was administered as a 24 hour therapy with 3 tablets. Each tablet contains 100 mg artesunate +250 mg sulfamethoxypyrazine/12.5 mg pyrimethamine. This drug is now being introduced in many African countries for the treatment of malaria. Doses administered in this trial were equal to the doses that are used for the treatment of malaria. The drug's safety and efficacy for treating malaria has been demonstrated in several studies, most recently in a large multicenter trial carried out in 4 different regions throughout Africa [**18**]. Due to the established safety profile, simplicity (one fixed dose combination tablet) and short administration time (24 hours) of Co-Arinate FDC®, the choice for using this ACT in the trial is justified. PZQ, being the first line standard treatment for schistosomiasis infection in Mali, was administered at the standard dose of 40 mg/kg.

### Laboratory procedures

At screening, each child provided one urine sample. A second sample was collected the following day before inclusion in the trial. Samples were taken between 10 am and 2 pm, and children were asked to run 3 laps before donating their samples. Appearance (macroscopic aspect) of the urine samples was reported and haematuria was checked (Hemastix®, Siemens Diagnostics, Belgium). At screening and at day 28 blood were obtained to measure White Blood cell Count 10^3^/µL (WBC), Red Blood cell Count 10^3^/µL (RBC), Haemoglobine g/dl (Hgb), Haematocrit % (Ht), Mean Corpuscular Volume fl (MCV), Mean Corpuscular Haemoglobin Concentration g/dl (MCHC), Mean Corpuscular Haemoglobin pg (MCH), Platelets 10^3^/µL, Lymphocytes 10^3^/µL, Alanine Aminotransferase U/L (ALT), Aspartate Aminotransferase U/L (AST) and creatinine µM/L).

Quantitative urine analysis was performed, using a filter technique with Whatman® filters. Two separate microscope filters were prepared for each sample, and evaluated by 2 independent, experienced microscopists. Data quality control for inter-observer variability was addressed by re-reading 10% of all the slides, picked out at random. If the first reading was positive and the second reading positive with a difference of less than 20%, mean number of eggs per 10 mL urine was calculated. If there was a difference between the two readers of 20% or more, a third reader was assigned to re-read the slides.

Infected children who were excluded from the study due to any of the above mentioned exclusion criteria, were treated at a standard recommended dose of 40 mg/kg PZQ.

Children were asked to come back and visit the team for treatment evaluation on 28 and 29 days after drug administration. Children who were not present on the evaluation days were actively searched for and brought to the centre by local guides. Two urine samples were collected from each child. If eggs were still present in these urine samples, a viability check was carried out. This test consists of checking for moving organelles of the miracidium inside the egg shell, hatching miracidia (in case of rupture of the egg shell) or beating of flame cellular. All dark eggs were considered as non viable. If all eggs in a sample were determined to be non viable (dark eggs or eggs without any of the viability characteristic listed above), the samples were considered to be egg negative.

### Outcome measurements

Study primary outcome was measured by treatment cure rate and egg reduction rate. Egg reduction rate was calculated as: [1 - (geometric mean of egg counts per 10 mL of urine after treatment divided by geometric mean of egg counts per 10 mL of urine before treatment)] ×100. Secondary outcomes were measured by (1) changes in urine appearance (before mixing the urine sample with the staining solution, the visual appearance of each urine sample was noted as being clear, cloudy or blood stained) before and after treatment, (2) changes in haematuria (using Hemastix®) before and after treatment and (3) patients' opinion towards the different treatment arms. The patient's opinion on the treatment was evaluated by using a short questionnaire. All adverse events were recorded following the days of treatment, all children were invited to come to the centre for two days of active follow-up, as well as at any time during the study period for adverse events (sign or symptoms). Investigators were permanently present at the study site. Adverse events are considered drug related if the same adverse events were not reported at the first presentation to the clinic during screening and the clinician did not find another explanation.

### Data management and statistical analysis

Data were double entered, validated using Microsoft ACCESS and analyzed with SPSS 12.0 for Windows. The intention-to-treat (ITT) analysis included all the randomized patients, excluding one case who received both investigational drugs. The ITT analysis was used for analysis of baseline comparison and adverse events. Per-protocol (PP) analysis was used for baseline comparison and for efficacy evaluation. Pearson Chi-square test, Yates Chi-square test or Fisher Exact tests were used to compare categorical variables. Mann-Whitney U test and Unpaired Student t-test were used to assess differences between treatment arms in the means for continuous variables. The McNemar paired chi-square test was used to compare the frequency of haematuria and the proportion of biological parameter normal values before and after treatment in each treatment arm. Wilcoxon T test was used to compare laboratory values before and after treatment administration.

## Results

**Figure 1:**
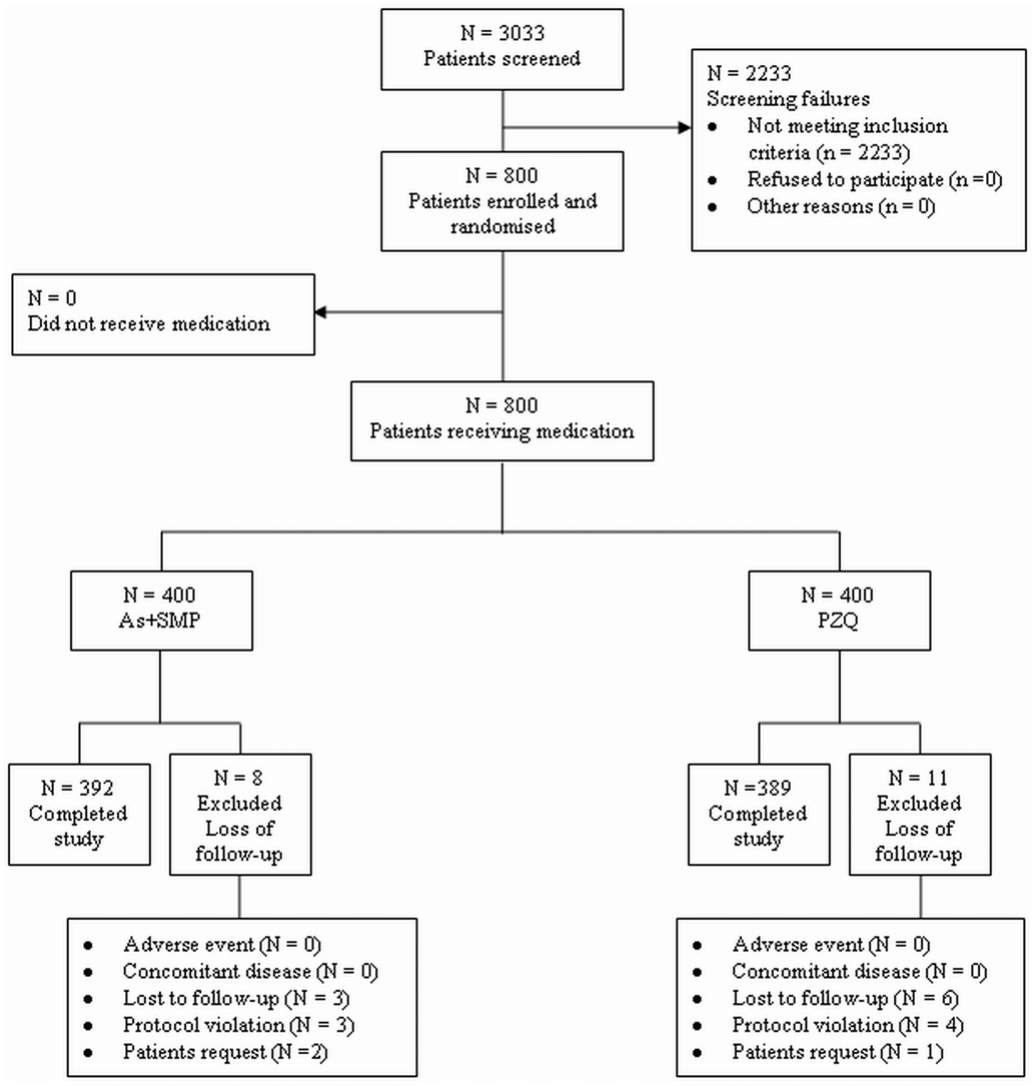
Trial profile. Out of a total of 3033 children (age range 6–15 years) screened for infection, 2233 children did not meet inclusion. A total of 392 children completed the study in the As/SMP treatment arm. Three children were lost to follow-up, in 3 cases the protocol was violated, and 2 patients chose to withdraw from the study. One patient assigned to the PZQ treatment arm received both medications and was withdrawn from analysis. Six children were lost to follow-up, 1 patient voluntarily withdrew from the study, and the protocol was violated in 4 children. A total of 389 children completed the study in the PZQ treatment arm (Figure 1).

Out of a total of 3033 children (age range 6–15 years) screened for infection, 2233 children did not meet our inclusion criteria. A total of 392 children completed the study in the As+SMP treatment arm. Three children were lost to follow-up, in 3 other cases the protocol was violated, and 2 patients chose to withdraw from the study. In the PZQ treatment arm, one patient received both medications and was withdrawn from analysis. Six children were lost to follow-up, 1 patient voluntarily withdrew from the study, and the protocol was violated in 4 children. A total of 389 children completed the study in the PZQ treatment arm (**Figure 1**).

### Demographics

At enrolment, the two study treatment groups were similar with regard to age, sex, ethnicity, weight, height and egg count per 10 mL of urine, p>0.05. The mean age was 10.45 years, and 35.8% of all the patients were female (**Table 1**).

**Table 1:** Baseline characteristics.

	As+SMP N = 392	PZQ N = 389
**Age**		
Mean Age ± SD	10.4±2.4	10.5±2.5
Range (Min, Max)	9.4 (6, 15.4)	9.2 (6, 15.2)
Median	10.2	10.5
**Sex**		
Sex Male	251	252
Sex Female	141	137
**Ethnicity**		
Bambara	211	220
Sarakole	41	30
Malinke	27	39
Peulh	24	23
Dogon	16	16
Senoufo	12	11
Dafing	11	13
Bobo	16	7
Sonrhaï	13	14
Kakolo	8	5
Others	13	11
**Weight**		
Mean Weight ± SD	27.7±7.5	28.0±7.3
Range (Min, Max)	33 (17, 50)	34 (16, 50)
Median	26.0	27
**Height**		
Mean Height ± SD	133.5±12.5	133.7±11.8
Range (Min, Max)	58 (108, 166)	61 (108, 169)
Median	133.0	133.0
**Eggs count Day 0** *		
Geometric Mean Eggs	40.9	41.4
Range (Min, Max)	1458 (2, 1460)	1382 (2, 1384)
Median	42.2	41.2
**Haematuria detected by Hematix**		
Haematuria +	324	339
Haematuria −	66	48
Total	390	387
**Urine macroscopic aspect**		
Hematic +	160	177
Hematic −	232	212
Total	392	389

* Mann-Whitney U test.

### Efficacy

#### Urine aspect (data not shown)

Microscopic haematuria and macroscopic aspect of urine did not significantly differ between the two treatment groups (Per protocol analysis) before as well as after the treatment with PZQ or As+SMP.

#### Egg counts per 10 mL of urine

The geometric mean of egg count in each group before treatment was comparable 42.2 in As+SMP group and 41.2 in PZQ group (Table 1). In terms of presence of eggs in urine after treatment, the cure rate in the PZQ group was 53%. After correction for egg viability, the cure rate was determined to be 97.7%. In the As+SMP treatment group, the cure rate was 43.9% and 81.4% after correction for egg viability. The cure rates before and after egg viability testing were higher in the PZQ group than in the As+SMP group, respectively Chi-square  = 6.44, p-value  = 0.011 and Chi-square  = 55.26, p-value <0.001 (Table 2). After treatment the geometric mean of egg counts without viability test was 2.9 under As+SMP versus 1.8 under PZQ, Mann-Whitney U test p<0.001.

**Table 2:** Repartition of cure rate by treatment arm before and after viability test on day 28.

	As+SMP	PZQ
	without viability testing	with viability testing	without viability testing	with viability testing
–*S. haematobium* negative children	172	319	206	380
*S. haematobium* positive children	220	73	183	9
Cure Rate %	43.9	81.4	53.0	97.7
Total number of children treated	392		389	

The egg reduction rate without viability test under PZQ treatment was 95.6% in comparison to 92.8% under As+SMP treatment Chi-square  = 2.76, p = 0.096.

The geometric mean of egg counts after viability test was comparable: 1.1 for As+SMP versus 1.0 for PZQ. The egg reduction rate after viability test under PZQ treatment was 97.6% in comparison to 97.4% under As+SMP treatment, Chi-square  = 0.05, p = 0.829.

*Haematuria*: The frequency of haematuria on day 28 was 49.7% (193/388) under PZQ treatment in comparison to 57.5% (225/391) under As+SMP treatment, Chi-square = 4.77, p-value = 0.028. Within each treatment arm, haematuria was less frequent in children on day 28 in comparison to day 0 (57.5% (225/391) *versus* 83.1% (324/390) for As+SMP treatment group and 49.7% (193/388) *versus* 87.6% (339/387) for PZQ), p<10^−6^.

#### Patient opinion (data not shown)

Both treatments were found to be easy to use and patient opinion on the number of tablets to be taken, treatment intake and administration of drugs between the two treatments did not vary significantly between the two treatment arms.

### Safety

#### Adverse events

After treatment, the proportion of participants who experienced adverse events related to medication was 0.5% (2/400) in the As+SMP treatment arm versus 2.3% (9/399) in the PZQ group, Chi-square = 4.53, p-value = 0.033. Abdominal pain and vomiting were the most frequent adverse events for both treatment arms. Dizziness and headache were also observed in the PZQ group. All adverse events were mild (**Table 3**).

**Table 3:** Number of adverse events per treatment group. (R = related, NR = Not related, Tot = total).

	As+SMP	PZQ
	R	NR	Tot	R	NR	Tot
**Abdominal pain**	1	73	74	1	49	50
**Dizziness**	0	17	17	3	17	20
**Headache**	0	61	61	1	62	63
**Vomiting**	1	104	105	4	30	34
**Anal pruritus**	0	1	1	0	4	4
**Anorexia**	0	3	3	0	0	0
**Boil**	0	1	1	0	0	0
**Chills**	0	1	1	0	1	1
**Convulsions**	0	1	1	0	0	0
**Cough**	0	28	28	0	24	24
**Conjunctivitis**	0	1	1	0	0	0
**Dermatosis**	0	4	4	0	4	4
**Diarrhoea**	0	9	9	0	8	8
**Hypersalivation**	0	1	1	0	0	0
**Local skin infections**	0	1	1	0	1	1
**Nausea**	0	7	7	0	5	5
**Otitis**	0	1	1	0	1	1
**Palor**	0	3	3	0	1	1
**Pruritus**	0	0	0	0	2	2
**Rhinobronchitis**	0	1	1	0	0	0
**Rhinorrhea**	0	12	12	0	9	9
**Sore throat**	0	1	1	0	2	2
**Thoracic pain**	0	3	3	0	5	5
**Urinary burn**	0	1	1	0	1	1
**Wound**	0	4	4	0	7	7
**TOTAL**	**2**	**339**	**341**	**9**	**233**	**242**

#### Laboratory evaluation

Comparison of biological parameters between the two treatment arms shows that all the parameters (White Blood cell Count 10^3^/µL (WBC), Red Blood cell Count 10^3^/µL (RBC), Haemoglobine g/dl (Hgb), Haematocrit % (Ht), Mean Corpuscular Volume fl (MCV), Mean Corpuscular Haemoglobin Concentration g/dl (MCHC), Mean Corpuscular Haemoglobin pg (MCH), Platelets 10^3^/µL, Lymphocytes 10^3^/µL, Alanine Aminotransferase U/L (ALT), Aspartate Aminotransferase U/L (AST) and creatinine µM/L) were similar on day 0. After treatment (Day 28) only the mean WBC increased significantly under As+SMP treatment (7.7±2.6 in As+SMP, versus 7.1±2.0 in the PZQ group), p = 0.007.

**Table 4** indicates that, within the As+SMP group, the mean WBC, MCHC and creatinine levels increased significantly over time, while the mean RBC, Ht, MCV, Platelets and ALT decreased significantly after treatment. Hgb, MCH, lymphocyte count and AST levels remained stable. Within the PZQ group, the mean MCHC, MCH, AST and creatinine values increased significantly in comparison to the mean values of WBC, RBC, Ht, Platelet counts, and ALT, which decreased significantly. Hgb, MCV and lymphocyte counts did not change after treatment.

**Table 4:** Evaluation of laboratory values over time (Biological Tolerance).

	As+SMP	PZQ	p*
	Mean±SD	Med	Min Max	NAV	Mean±SD	Med	Min Max	NAV	
**WBC (10^3^/µL)**									
Day 0	7.3±2.1	6.9	3.4, 17.6	46	7.3±2.1	7	2.8, 18.0	43	*0.622*
Day 28	7.7±2.6	7.2	3.0, 17.9	78	7.1±2.0	6.8	3.1, 20.6	43	*0.007*
**p** **	*0.005*				*0.005*				
**RBC (10^3^/µL)**									
Day 0	4.5±1.1	4.4	2.8, 24.0	26	4.4±0.5	4.4	1.8, 6.9	24	*0.675*
Day 28	4.4±0.4	4.4	3.2, 5.7	15	4.4±0.4	4.4	3.0, 6.1	22	*0.628*
**p** **	*0.025*				*0.005*				
**Hgb (g/dl)**									
Day 0	11.2±1.4	11.4	7.4, 13.9	64	11.1±1.5	11.4	3.7, 14.3	63	*0.746*
Day 28	11.4±0.9	11.4	7.9, 14.5	18	11.3±0.9	11.4	7.4, 13.4	17	*0.981*
**p** **	*0.488*				*0.376*				
**Ht (%)**									
Day 0	35.5±3.1	35.6	25.3, 44.3	21	35.3±3.3	35.6	14.9, 43.0	18	*0.57*
Day 28	35.1±2.6	35.3	27.3, 42.6	10	34.9±2.6	35	26.9, 41.8	11	*0.537*
**p** **	*<0.001*				*0.001*				
**MCV (fL)**									
Day 0	80.8±5.7	80.8	60.9, 98.2	20	80.3±6.4	81.2	57.1, 99.2	38	*0.568*
Day 28	80.2±5.6	80.5	60.0, 94.0	23	80.2±6.0	81.1	56.9, 95.5	32	*0.706*
**p** **	*0.011*				*0.339*				
**MCHC (g/dl)**									
Day 0	31.5±2.8	32.3	21.7, 35.5	100	31.4±3.1	32.2	21.1, 50.3	102	0.61
Day 28	32.3±1.2	32.4	26.3, 36.1	33	32.4±1.2	32.4	26.4, 35.4	31	0.486
**p** **	0.038				<0.001				
**MCH (pg)**									
Day 0	25.4±3.0	25.9	14.9, 31.7	60	25.3±3.5	26	13.1, 41.7	73	0.827
Day 28	25.9±2.4	26.3	15.8, 39.1	27	25.9±2.4	26.4	16.8, 31.2	33	0.557
**p** **	0.527				0.003				
**Platelets (10^3^/µL)**									
Day 0	364.2±90.2	359	36.0, 804.0	53	363.3±96.7	354	142.0, 1000	54	0.522
Day 28	338.6±85.6	330	94.0, 799.0	30	347.6±91.2	338.5	83.0, 965.0	46	0.206
**p** **	<0.001				<0.001				
**Lymphocytes (10^3^/µL)**									
Day 0	2.9±0.8	2.7	1.2, 6.3		2.8±0.8	2.8	1.2, 7.3		0.911
Day 28	2.9±0.8	2.8	1.3, 5.6		2.9±1.0	2.8	1.1, 14.1		0.403
**p** **	0.63				0.169				
**ALT (U/L)**									
Day 0	29.8±16.5	25	6.0, 146.3	31	28.8±15.8	23.7	9.1, 118.5	31	0.172
Day 28	26.4±17.3	23.1	6.2, 274.7	14	26.1±13.6	22.9	3.6, 138.7	18	0.682
**p** **	<0.001				0.004				
**AST (U/L)**									
Day 0	43.1±26.6	38.4	15.2, 451.0		40.4±22.3	36.3	9.7, 398.0		0.109
Day 28	43.9±20.9	39.3	14.7, 194.2		44.3±20.8	39.1	9.9, 173.6		0.973
**p** **	0.165				0.016				
**Creatinin (µM/L)**									
Day 0	75.8±34.1	72	10.3, 621.0	374	73.7±18.9	70.9	37.2, 148.1	377	0.52
Day 28	84.7±24.6	82.3	26.0, 313.1	385	86.9±26.8	83.2	35.8, 228.9	383	0.59
**p** **	<0.001				<0.001				

* **Mann-Witney U test.**

** **Wilcoxon T test for paired samples.**

## Discussion

The efficacy and safety of the antimalarial ACT As+SMP, administered in the doses used for the treatment of malaria, were compared to the efficacy and safety of PZQ, the standard treatment for schistosomiasis, in children aged 6 to 15 years. As+SMP induced a egg reduction rate of 92.8% compared to 95.6% obtained with PZQ. The cure rate without viability test was 43.9% in the As+SMP group versus 53% in the PZQ group (Chi^2^ = 6.44, p = 0.011), meaning that PZQ was more effective than As+SMP for treating *S. haematobium* infections. The cure rate, based on the absence of viable eggs, was 81.4% in the As+SMP group versus 97.7% in the PZQ group (Chi^2^  = 55.26, p<0.001). However, the latter 2 figures have to be interpreted with care, since the viability test was used only after completion of treatment in the two groups, and a control group was not included in the analysis. In addition, the viability test, as performed in this study, is not routinely used for diagnosis and treatment evaluation, and to date has never been used in drug evaluation trials in *S. haematobium* infected patients [**19**]. The presence of viable eggs in the urine corresponds to an active infection, which may be due to reduced strain susceptibility to the drug, questions about drug quality, drug bioavailability, to high initial intensity of infection or to pre-patent infection at the time of screening [**4**]. For example, for PZQ, studies in which the recommended doses were used have recorded cure rates from 60% to 90% for *S. haematobium* infections [**20]**, [21], [22****]. In addition, PZQ has reduced *S. haematobium* worm burdens in a moderately endemic situation in Burundi by 94% [**23**]. Finally, a recent study in Zimbabwe showed a cure rate of 88.5% with PZQ treating *S. haematobium*-infected persons [**24**].

In this study, PZQ was more efficacious to reduce haematuria during *S. haematobium* infections in comparison to As+SMP, administered in doses used for the treatment of malaria. The repartition of haematuria on day 28 between the two groups, as determined by the Hemastix® test, shows a statistical difference (49.7% under PZQ treatment versus 57.5% under As+SMP, Chi-square = 4.77, p = 0.028). This difference is most likely related to the difference in cure rate observed between the two treatment arms.

The safety and tolerability profile of As+SMP was similar to the profile seen with PZQ. All clinical adverse events were graded mild and were resolved during the 2 active follow-up days. No serious adverse event was recorded with any one of the treatments administered, as documented in previous studies [**21]**, [25], [26], [27], [28], [29****]. Our study shows that abdominal pain and vomiting were the most frequent adverse events for both treatment arms. Related adverse events were more frequent in the PZQ group than in the As+SMP group, p-value  = 0.033. Previous studies have observed similar adverse events in patients who were being treated with praziquantel [**4]**, [24], [30], [31], [32****].

Overall, the means of all laboratory parameters were normal on days 0 and 28, except for the creatinine for both treatment arms. This can be explained by the effect of *S. haematobium* on the kidneys [**33]**, [34], [35****].

### Conclusion

Although the safety and tolerability profiles of both treatment arms are similar, PZQ was more effective than As+SMP in this clinical set up. However, the doses of As+SMP that were used in this trial are doses commonly used for the treatment of malaria. Further research into correct dosing for this specific indication may be recommendable. It will be interesting to evaluate the efficacy and safety of As+SMP on other *Schistosoma* species, such as *Schistosoma mansoni,* as well as the effect of the combination of As+SMP and PZQ on both *S. haematobium* and *S. mansoni*.

## Supporting Information

Checklist S1 CONSORT Checklist
(0.07 MB DOC)

Protocol S1 Trial Protocol
(0.32 MB DOC)
