# Supplementary material for: Efficacy of Artesunate + Sulfamethoxypyrazine/Pyrimethamine versus Praziquantel in the Treatment of Schistosoma haematobium in Children
Source: PLoS One. 2009 Oct 5;4(10):e6732. doi: 10.1371/journal.pone.0006732 (PMC2749939; doi:10.1371/journal.pone.0006732)
Supplement: Protocol S1 — Trial Protocol (0.32 MB DOC) [file pone.0006732.s002.doc]

**PROTOCOLE**

**Code d’étude:** 2007/S4

**Titre:**

**Essai clinique randomisé double aveugle comparant l’efficacité de l’artesunate + sulfamethoxypyrazine/pyrimethamine versus praziquantel dans le traitement de la bilharziose urinaire chez les enfants.**

**Type d’étude: Essai clinique randomise double aveugle**

**Période d’étude: Juillet 2007 – Décembre 2007**

| **Centre d’étude** |  | **Promoteur** |
| --- | --- | --- |
| MRTC/DEAP/FMPOS  Université de Bamako  BP 1805, Bamako  Mali  Tel: +223 222 81 09  Fax: +223 222 81 09  [okd@mrtcbko.org](mailto:okd@mrtcbko.org) |  | Dafra Pharma nv  Slachthuisstraat 30/7  2300 Turnhout  Belgium  Tel: +32 14 61 78 20  Fax: +32 14 61 78 59  info@dafra.be |
| **Investigateur Principal** |  | **Représentant du Promoteur** |
| Mahamadou Soumana Sissoko |  | Sarah Cauwenbergh |
| **Directeur du Centre** |  | **Directeur du Centre** |
| Pr. Ogobara Doumbo |  | Bruno Jansen |

**INVESTIGATEURS ET LEURS DECLARATIONS**

Nous les soussignés, avons lu et compris ce protocole et par conséquent consentons à conduire l'étude conformément à ce protocole et à accomplir toutes les exigences concernant les obligations d'investigateurs. L’étude prendra en outre compte de toutes les autres exigences pertinentes des Directives ICH (Conférence Internationale sur l’Harmonisation) pour une bonne pratique clinique aussi bien que les exigences de la Déclaration de Helsinki (Tokyo 2004), les Lois Nationales et les Règlements. Nous nous conformerons à tous les modes opératoires normalisés exigés pour la conduite de cette étude.

| **Centre d’étude** | **________________________** Date :______/______/_______  **Ogobara Doumbo, MD, PhD** D D M M AA  Directeur  _________________________ Date: ______/______/_______  **Mahamadou S. Sissoko, MD, MSPH** D D M M AA  Investigateur Principal  __________________________ Date: ______/______/_______  **Abdoulaye Dabo, MSc, PhD** D D M M AA  Co-investigateur |
| --- | --- |

**PROMOTEURS ET LEURS DECLARATIONS**

Nous les soussignés, avons préparé, lu et compris ce protocole et par conséquent, consentons à conduire l'étude conformément à ce protocole et à accomplir toutes les exigences concernant les obligations de promoteurs. Nous nous engageons en outre à remplir toutes les autres exigences pertinentes des Directives ICH (Conférence Internationale d’Harmonisation) pour une bonne pratique clinique aussi bien que les exigences de la Déclaration de Helsinki (Tokyo 2004), les Lois Nationales et les Règlements. Nous nous conformerons pour cela à tous les modes opératoires normalisés exigés pour la conduite de cette étude.

| **Sponsor** | _______________________ Date :______/______/_______  **Bruno Jansen** D D M M AA  Directeur  ________________________ Date :______/______/_______  **FH Jansen** , **M.D., Ph. D.** D D M M AA  Président  Directeur R&D  ________________________ Date: ______/______/_______  **Sarah Cauwenbergh** , **MSc** D D M M Y Y  Représentant du Promoteur |
| --- | --- |
|  |  |

**APPROBATION DU PROTOCOLE**

**Code d’étude**: 2007/S4

Nous reconnaissons que les renseignements relatifs à cette étude et le médicament fourni par le promoteur n'ont pas été publiés précédemment et demeurent confidentiels.

Nous acceptons que tout changement porté au protocole soit auparavant approuvé par écrit par le promoteur et le Comité d'Éthique Indépendant avant sa mise en œuvre (excepté le cas des changements qui nécessitent la protection et la sécurité des participants ou quand les changements impliquent seulement les aspects administratifs de l'étude).

Nous donnons notre consentement pour conduire l'étude conformément à ce protocole. Nous nous conformerons à toutes les exigences de la version courante de la Déclaration de Helsinki, les Directives courantes ICH - GCP (Conférence Internationale d’Harmonisation - Bonne Pratique Clinique), les Lois Nationales pertinentes et les Règlements. Nous nous conformerons aux modes opérationnels normalisés recommandés pour la conduite de cette étude et assurerons que tous les autres partenaires impliqués dans cette étude en seront informés en fonction de leurs obligations.

**Investigateur Principal**: ______________________ ___/___/___

Mahamadou S. Sissoko Date

MRTC/DEAP/FMPOS

BP 1805, Bamako, Mali

Tel/Fax: +223 222 81 09

mssissoko@mrtcbko.org

**TABLE DES MATIERES**

[Investigateurs et leurs déclarations 2](#__RefHeading___Toc169436124)

[Promoteurs et leurs déclarations 3](#__RefHeading___Toc169436125)

[Approbation du Protocole 4](#__RefHeading___Toc169436126)

[1. Liste des abréviations 7](#__RefHeading___Toc169436127)

[2. Justification de l’étude 8](#__RefHeading___Toc169436128)

[3. OBJECTIFS 10](#__RefHeading___Toc169436129)

[3.1 Objectifs Primaires 10](#__RefHeading___Toc169436130)

[2.2 Objectifs Secondaires 10](#__RefHeading___Toc169436131)

[4. Methodologie 12](#__RefHeading___Toc169436132)

[4.1 Site d’étude 12](#__RefHeading___Toc169436133)

[4.2 Période d’étude 12](#__RefHeading___Toc169436134)

[4.3 Type d’étude 12](#__RefHeading___Toc169436135)

[4.4 Médicaments 13](#__RefHeading___Toc169436136)

[4.5 Population d’étude 14](#__RefHeading___Toc169436137)

[Critères d’inclusion 14](#__RefHeading___Toc169436138)

[Critères de non inclusion 14](#__RefHeading___Toc169436139)

[4.6 Arrêt du traitement 15](#__RefHeading___Toc169436140)

[4.6.1 Participation à l’étude 15](#__RefHeading___Toc169436141)

[4.6.2 Retrait du suivi et classification 15](#__RefHeading___Toc169436142)

[4.7 Résultats 16](#__RefHeading___Toc169436143)

[4.7.1 Résultats Primaires 16](#__RefHeading___Toc169436144)

[4.7.2 Résultats Secondaires 16](#__RefHeading___Toc169436145)

[4.8 Taille d’échantillon 16](#__RefHeading___Toc169436146)

[4.9 Procedure d’étude 17](#__RefHeading___Toc169436147)

[4.9.1 Recrutement et inclusion des patients 17](#__RefHeading___Toc169436148)

[4.9.2 Traitement 19](#__RefHeading___Toc169436149)

4.9.2.1 Médicaments ...…....………………………………………………………..19

4.9.2.2 Médicaments concomitants ...………………………………………………20

[4.9.3 Poursuite du traitement 20](#__RefHeading___Toc169436150)

[4.9.4 Examens de laboratoire 23](#__RefHeading___Toc169436151)

[Recherche Parasitologique 23](#__RefHeading___Toc169436152)

[Detection de l’hematurie 24](#__RefHeading___Toc169436155)

[Hématologie et Biochimie 25](#__RefHeading___Toc169436156)

[4.9.5 Plan de Supervision 25](#__RefHeading___Toc169436157)

[4.10 Considérations Ethiques et déontologiques……………………………….25](#__RefHeading___Toc169436158)

[4.10.1 Plan de soins des patients 26](#__RefHeading___Toc169436159)

[Echantillon d’urine………………………………………………………………….26](#__RefHeading___Toc169436160)

[Arrêt du traitement 26](#__RefHeading___Toc169436161)

[4.10.2 Confidentialité des résultats 26](#__RefHeading___Toc169436162)

[4.10.3 Risques potentiels et effets indésirables 27](#__RefHeading___Toc169436165)

[4.10.4 Risques et minimisation 27](#__RefHeading___Toc169436166)

[4.10.5 Risques, bénéfices et compensation 28](#__RefHeading___Toc169436167)

[4.10.6 Publications des résultats 28](#__RefHeading___Toc169436168)

[5. Références 29](#__RefHeading___Toc169436169)

[Annexe A : Consentement éclairé pour parent ou tuteur 31](#__RefHeading___Toc169436170)

Annexe B: Budget……………………………………………………………………..36

Annexe C: Curriculum Vitae des Chercheurs…………………………………………38

**1. LISTE DES ABREVIATIONS**

AS Artesunate

CRF Case Report Form (Fiche de Report de Cas)

epg Eggs per Gram (Oeufs par Gramme)

FDC Fixed Dose Combination (Combinaison fixe)

GCP Good Clinical Practice (Bonnes Pratiques Cliniques)

GLP Good Laboratory Practice (Bonnes Pratiques de Laboratoire)

GM Geometric Mean (Moyenne Géométrique)

GMP Good Manufacturing Practice (Bonne Pratique de Fabrication)

PI Principal Investigator (Investigateur Principal)

PZQ Praziquantel

SMP Sulfamethoxypyrazine/Pyrimethamine

SOP Standard Operation Procedure (Mode Opératoire Normalisé)

WHO World Health Organization (Organisation Mondiale de la Santé)

**2. JUSTIFICATION DE L’ETUDE**

Environ 200 millions de personnes dans le monde sont infectées par la bilharziose, une maladie parasitaire tropicale, causée par un trématode appartenant au genre *Schistosoma*. Cent vingt millions de personnes présentent des symptômes, et parmi eux 20 millions font une maladie sévère. Quatre vingt cinq pour cent des personnes infectées vivent en Afrique au sud du Sahara, ce qui fait que cette maladie parasitaire soit la plus importante de cette région après le paludisme (1,2). Il y’a 5 espèces de schistosomes qui infectent l’homme (*Schistosoma mansoni, S. intercalatum, S. japonicum, S. mekongi et S. hæmatobium*). Parmi ces espèces, *S. mansoni* et *S. haematobium* sont les plus fréquemment rencontrées en Afrique. Notre étude portera exclusivement sur *S. haematobium*.

Le cycle de transmission de la bilharziose passe par la contamination de l’eau de surface par les excrétas (selles et urine) contenant les œufs de schistosome. Les cours d’eau naturels (rivières, lacs, mares, fleuves, étangs) sont les principales sources de contamination. Toutefois, les sources d'eau artificielles ont récemment contribué à la propagation de la bilharziose. Les œufs excrétés libèrent un miracidium qui infecte l’espèce de mollusque hôte intermédiaire qui lui est spécifique. A l’intérieur de cet hôte, le développement et la multiplication asexuée du miracidium conduisent à la formation des cercaires qui sont ensuite libérées dans l'eau. Les schistosomes sont des helminthes qui parasitent les hôtes définitifs (homme et animaux) par la pénétration transcutanée active des cercaires à l’origine de démangeaisons. Les schistosomules sont ensuite véhiculés à travers le cœur et les poumons, s'installent temporairement au niveau de la veine porte où ils grandissent et s’accouplent. Au moment de la ponte, ils migrent au niveau de la paroi périvésicale (*S. hæmatobium*) ou au niveau du plexus mésentérique de la veine porte (*S. mansoni*), où a lieu l'oviposition. Une partie des œufs pondus sont alors éliminés avec les excrétas et le cycle de transmission recommence (3). En rapport avec la localisation et le nombre d’œufs pondus par les femelles, les réactions immunologiques des hôtes contre les œufs et leurs antigènes, une série de symptômes aigus et chroniques peuvent apparaître. La fièvre de Katayama, la formation de granulome, la diarrhée, l’hématurie, l’ulcère, l’anémie, la fibrose de Smear, le retard de la croissance et du développement cognitif, la splénomégalie, l’hépatomégalie, le cancer de la vessie et une atteinte sévère du rein sont entre autres quelques-uns des symptômes et complications qui peuvent s’observer chez les malades (3). Les symptômes et les séquelles causés par les schistosomes sont d’autant plus sévères que la morbidité est élevée,

Le traitement et le contrôle de la maladie sont une nécessité. Le Praziquantel (PZQ) utilisé au Mali depuis les années 1970 est le médicament de choix pour traiter la bilharziose. Cependant ce médicament n'est pas efficace contre les stades immatures et les jeunes parasites. De plus, le problème de la résistance du parasite et la rapidité de la réinfection nécessitent le développement de nouveaux médicaments efficaces (1). La plante médicinale *Artemisia annua* a été utilisée en Chine pendant plus de 2000 ans pour traiter toutes les helminthiases (4). Pendant les dernières décennies, l’artemisinine, le principe actif de la plante et ses dérivés a été découvert. Récemment, il a été utilisé efficacement dans le traitement des infections palustres, habituellement en combinaison avec un autre antipaludique pour prévenir le développement de la résistance des parasites. Des études ont été conduites sur l'utilisation des dérivés de l'artemisinine dans le traitement des schistosomes. L'artesunate adminsitré en monothérapie dans le traitement de différentes espèces de schistosome s’est révélé inefficace (11,12). En plus, la plupart des cas de bilharziose se trouvent dans les régions souffrant de paludisme. L’utilisation exclusive des dérivés de l'artemisinine (ou en combinaison avec le PZQ) dans ces régions endémiques n'est pas efficiente, parce que cela équivaudrait à une monothérapie notamment pour les malades coinfectés par le paludisme et la bilharziose. Par conséquent, il est important que des combinaisons efficaces dans le traitement du paludisme tels les dérivés de l'artemisinine soient aussi étudiées pour évaluer leur effet sur la bilharziose comme s’est le cas dans cette étude. Les études préliminaires avec la combinaison Artesunate plus Sulfamethoxypyrazine-Pyrimethamine (As+SMP) ont donné des résultats satisfaisants dans le traitement de *S. mansoni* aussi bien sur les stades matures que sur les jeunes parasites (4,6). Une autre étude menée par Adam et *al*. (en 2007) a montré l’efficacité des combinaisons thérapeutiques à base d’artemisinine (CTA) contre la bilharziose quand elles sont administrées dans le traitement du paludisme non compliqué (13). Les CTA pourraient être combinées avec le PZQ en ciblant donc les différents stades de développement du parasite pour améliorer les résultats du traitement. L’évaluation de l’efficacité des dérivés de l’artemisinine dans le traitement des schistosomes revêt donc une grande importance. Cela se comprend aisément par le fait que ces produits sont couramment utilisés contre le paludisme, et ensuite parce que le paludisme et la bilharziose sont deux affections à la fois endémiques dans les mêmes régions. Par ailleurs, selon Inyang-Etoh et *al*., (en 2004), l’artesunate serait plus rentable que le PZQ en terme de coût/efficacité (5). Le praziquantel (PZQ) reste encore le médicament de choix, le seul qui soit efficace sur toutes les espèces de schistosome. En raison de l'inquiétude grandissante de la résistance du parasite au PZQ, il est temps d’imaginer d’autres alternatives.

Notre étude a donc pour but d’évaluer l’efficacité de la combinaison As+SMP (Co - Arinate FDC ®) comparée à celle du PZQ dans le traitement de *S. haematobium* chez les enfants vivant dans le district de Bamako, Mali.

**3. OBJECTIFS**

Le but de cette étude est d’évaluer l’efficacité du PZQ versus As+SMP (Co - Arinate FDC ®) dans le traitement des enfants maliens âgés de 6-15 ans infectés par *S. hæmatobium*.

L'hypothèse nulle dans cette étude sera que la combinaison As+SMP est plus efficace que le PZQ dans le traitement de l’infection due à *S. hæmatobium*.

**3.1 OBJECTIFS PRIMAIRES**

- Comparer le taux de guérison entre les bras de traitement.
- Comparer la charge parasitaire entre les bras de traitement.
- Comparer le niveau de production des œufs entre les bras de traitement.

**3.2 OBJECTIFS SECONDAIRES**

- Evaluer les changements relatifs à l’aspect des urines avant et après le traitement.
- Evaluer les changements relatifs à la fréquence de l’hématurie avant et après le traitement.
- Evaluer l'observance du traitement dans les deux bras de traitement.
- Evaluer les réactions adverses cliniques et biologiques entre les deux bras.

**4. METHODOLOGIE**

**4.1 SITE D’ETUDE**

Cette étude se déroulera à Djalakorodji quartier péri-urbain de Bamako, Mali. Le village situé au nord de la ville compte 28,000 habitants. Il est traversé par de nombreuses rivières temporaires le long desquelles se sont installées les populations. Ces rivières sont alimentées par les eaux de pluie et s’assèchent quelques mois après l’hivernage (mars-avril) selon la quantité de pluies enregistrées au cours de l’année. Pendant toute la période de mise en eau, ces cours d’eau sont utilisés par les populations au cours de plusieurs activités dont la lessive, la vaisselle, la pêche, les baignades et autres activités ludiques pour les enfants. Ils constituent à cet effet d’excellents gîtes à mollusques où s’infestent régulièrement les populations notamment les enfants. Les risques de contamination sont d’autant plus élevés que les sources d’approvisionnement en eau potable se réduisent essentiellement à ces points d’eau notamment pendant l’hivernage (Thèses Kourané et Saibou, Bamako, Mali).

**4.2 PERIODE D’ETUDE**

Le recrutement des malades commencera le 1er août et durera jusqu'au 30 août 2007. Cependant, si le nombre de malades requis n'est pas atteint dans le délai imparti, alors la période de l'inclusion pourrait être prolongée. Cependant, le Comité d'Ethique et le partenaire Dafra Pharma nv/sa seront informés d’avance de toute extension probable de la période d’étude. Tous les malades seront suivis pendant 28 jours (septembre 2007) après l’inclusion. La période d’évaluation après le suivi s’étendra sur un mois (octobre 2007). Nous consacrerons 3 mois (novembre 2007 à janvier 2007) à la gestion et à l’analyse des données.

**4.3 TYPE D’ETUDE**

Il s’agit d’un essai clinique randomisé double aveugle comparant l'efficacité de As+SMP versus le PZQ dans le traitement de la bilharziose chez les enfants âgés de 6 à 15 ans.

Le recrutement des enfants sera subordonné au fait qu’ils soient infectés par *S. hæmatobium*. Les enfants excréteurs d’œufs seront alors soumis au traitement soit avec l’As+SMP soit avec le PZQ de façon randomisée double aveugle.

**4.4. LES MEDICAMENTS**

PZQ et As+SMP (Co-Arinate® FDC) seront comparés:

1. **Produit 1**

Co-Arinate® FDC - Artesunate /Sulfamethoxypyrazine/Pyrimethamine

100 mg 250 mg 12.5 mg

Fabriqué par : Dafra Pharma nv

Forme du dosage : Comprimé

Formulation : Junior

BatchNumber : 07-F-18

Date de fabrication : 18/06/007

Date d’expiration: 06/2009

Dose : 4mg/kg artesunate seront administrés par

patient. Le guide d’administration en fonction

du poids du patient peut être trouvé dans

l’annexe G: Instructions pour le traitement.

1. **Produit 2**

Praziquantel - 400mg

Fabriqué par : Dafra Pharma nv

Forme du dosage : Comprimé

Formulation : Junior

BatchNumber : 25-F-07

Date de fabrication : 25/06/2007

Date d’expiration: 06/2009

Dose : 40mg/kg seront administrés par patient. Le

guide d’administration en fonction

du poids du patient peut être trouvé dans

l’annexe G: Instructions pour le traitement

**4.5. POPULATION D’ETUDE**

Les sujets de l'étude seront des enfants âgés de 6 à 15 ans identifiés dans la population générale. Ils seront recrutés à Djalakorodji, Bamako, Mali. Les sujets seront évalués pour leur éligibilité et subiront des examens physiques, clinique et de laboratoire afin de suivre la tolérance clinique et biologique due au médicament administré. L'éligibilité sera évaluée sur la base des critères d’inclusion et de non inclusion présentés ci-dessous.

***Critères d’inclusion***

Les sujets qui seront inclus dans l'étude doivent présenter les critères suivants:

- Etre âgés de 6 à 15 ans,
- Etre en bon état de santé selon l’avis du médecin de l'étude,
- Etre infectés par la bilharziose uro-génitale diagnostiquée par la présence des œufs de *S. haematobium* dans les urines,
- Etre résidants de Djalakorodji,
- Etre capables de prendre le médicament par la voie orale,
- Obtenir le consentement éclairé écrit du parent ou de la personne en charge de l’enfant pour sa participation à l'étude.

***Critères de non inclusion***

Les sujets ne seront pas inclus pour l’une des raisons suivantes:

- Avoir un poids supérieur à 50 kg,
- Etre enceinte ou allaitante au moment de l'étude,
- Etre atteint d’une maladie sévère sur la base de l’examen clinique telle que la cysticercose cérébrale, HIV, …
- Avoir des signes de malnutrition sévère (enfants ayant un rapport poids/taille en dessous de 3 déviations standards ou au-dessous de 70% de la médiane selon les valeurs de références standardisées de l’OMS ou encore avec œdème symétrique qui affecte les deux pieds),
- Avoir une hypersensibilité à l’As, à la SMP ou au PZQ,
- Avoir pris une autre antipaludique (CTA) ou un médicament anti-bilharzien pendant l'étude,
- Avoir participé à une étude antérieure du genre.

**4.6 ARRET DU TRAITEMENT**

Les résultats valides de l'étude porteront à la fois sur les enfants ayant fait l’objet d’un suivi complet et les non inclus de l’étude.

**4.6.1 Participation à l’étude**

Les malades qui ont participé à l’ensemble des étapes de l'étude sans être exclus au cours du suivi seront inclus dans l’analyse de l'échantillon. Les taux de guérison seront évalués à la fin de l'étude. Le taux de guérison sera exprimé par la proportion de malades n'excrétant pas d'œufs à J28 (28^ème^ jour).

**4.6.2 Retrait du suivi et classification**

Le taux de perdus de vue est supposé être nul, parce que nous rendrons visite à domicile aux malades qui ne se présenteraient pas aux différents examens. Dans le cas du déplacement inopiné d’un malade pendant la période de l'étude, celui-ci sera considéré comme perdu de vue et ne sera donc pas inclus dans l'analyse. S’il arrive qu’un malade reçoive le traitement qui n’est pas le sien, il ne sera pas retiré de l’étude, mais ses résultats seront analysés dans le groupe de traitement convenable.

Les critères objectifs justifiant l’arrêt du traitement et/ou l’administration d’un autre médicament sont:

- Le retrait du consentement du parent/personne en charge (retrait volontaire),
- Le développement d'une maladie concomitante qui perturberait l'interprétation des résultats de l'étude (retrait involontaire),
- L’incapacité de retenir le médicament à cause des vomissements (retrait involontaire),
- L’apparition de tout événement adverse sévère qui exige le retrait du traitement selon l’avis de l’investigateur ou à la demande du parent/personne en charge (retrait involontaire/volontaire),
- La violation du protocole: un malade sera retiré de l'étude si son recrutement et/ou le suivi n’ont pas été faits suivant les conditions du protocole. Par exemple, la discrétion de l'investigateur, l’omission de la dose du médicament ou un rapport crédible de prise d'antibilharziens supplémentaires ou encore l’utilisation de médicaments antipaludiques en dehors de ceux du protocole de l'étude (automédication). Tout évènement qui ne permettrait pas une interprétation correcte de la réponse au traitement sera considéré comme une violation du protocole.

**4.7 RESULTATS DE L’ETUDE**

**4.7.1 Résultats primaires**

Pour évaluer la réponse thérapeutique, le paramètre fondamental à mesurer est l’excrétion des œufs par les malades.

**4.7.2 Résultats secondaires**

Les autres paramètres peuvent être mesurés pour l’obtention des résultats thérapeutiques des sessions de traitement:

- - Changement relatif à l’aspect de l'urine avant et après traitement.
  - Changement relatif à la fréquence de l’hématurie avant et après traitement.
  - Observance du traitement par les malades.
  - Evènements adverses cliniques et biologiques.

**4.8 TAILLE D’ECHANTILLON**

Dans le quartier de Djalakorodji, la fréquence de *S. haematobium* chez les enfants est de 70,8%. Par conséquent, nous estimons qu’en examinant les urines de 1150 enfants à la recherche des excréteurs d’œufs de *S. haematobium*, nous détecterons 814 enfants infectés.

Pour calculer la taille de l'échantillon nous avons estimé le taux de guérison à 75% (60-95%) avec le praziquantel (11,12) et +10% avec As+SMP. Avec une puissance de 90% et une erreur de type alpha égale à 5% nous aurons besoin de 354 malades dans chacun des bras de traitement. Un nombre supplémentaire de 46 enfants sera ajouté dans chaque bras de traitement pour corriger les éventuels perdus de vue et la non observance du traitement par les malades. Le nombre total d'enfants nécessaire à l’étude sera de 800.

La randomisation par bloc (100 blocs, 8 malades par bloc) sera utilisée pour allouer les malades aux deux bras de traitement de manière à ce que les investigateurs ne sachent pas la nature du médicament que chaque malade recevra. La randomisation actuelle utilisera le système des enveloppes. L'enveloppe dissimule le traitement que le malade doit recevoir. Les enveloppes seront numérotées séquentiellement à l'extérieur et à l’intérieur et il aura soit la mention A/C ou B/D correspondant aux deux traitements.

La liste de randomisation contient le nombre séquentiel pré-spécifié. Sur ce document, le médecin aura besoin de la date et de l’heure auxquelles l'enveloppe a été ouverte, les initiales du malade, le sexe, la date de naissance et le code du traitement.

La liste de randomisation sera donnée au responsable de la gestion des données pour vérifier l'observance du protocole par les investigateurs.

**4.9 PROCEDURES DE L’ETUDE**

**4.9.1 Recrutement et inclusion des malades**

La population d’étude sera composée des enfants de Djalakorodji âgés de 6 à 15 ans. Les parents ou personnes en charge des enfants éligibles qui exprimeront la volonté de participer à l’étude seront référés à la clinique. A la clinique, ils recevront une explication détaillée de l'essai. Toutes les explications et les procédures du consentement éclairé seront données dans la langue locale du participant, le bamanan kan. Les guides locaux de Djalakorodji et les membres de l’équipe parlent tous correctement cette langue. Le consentement éclairé sera documenté en français sous forme écrite, soumis et approuvé par le Comité d’Ethique de la Faculté de Médecine, de Pharmacie et d’Odonto-Stomatologie, Université de Bamako, Mali. Il est possible qu'une partie des personnes en charge des enfants soit illettrée. La signature ou l’empreinte digitale d’un témoin sera alors exigée dans ce cas de figure. Chaque enfant recevra un numéro de dépistage et les renseignements démographiques de base seront collectés à partir de la fiche d’identification du malade. Le clinicien procédera à l’interrogatoire (historique médicale) et à l’examen clinique initial de tous les volontaires pendant le dépistage pour vérifier les critères d'inclusion et de non inclusion. Cet examen clinique initial consistera à faire : la réception, l’interrogatoire, l’évaluation du statut nutritionnel, la palpation, la pesée, la prise de la température axillaire, la détermination des modifications du foie et/ou de la rate en mesurant les extensions en dessous de la cage thoracique et la palpation du foie et de la rate pour déterminer la fermeté des organes. La taille et le poids seront mesurés à l’inclusion. Toutes les informations seront enregistrées dans le cahier de report de cas (CRF).

Deux (2) échantillons d'urine seront collectés sur 2 jours consécutifs. Les enfants excrétant des œufs de *S. haematobium* dans leur urine seront les seuls éligibles à participer à l'étude si les autres critères d'inclusion sont respectés. L'aspect des échantillons sera rapporté (couleur, consistance, présence de sang, trouble/clair). Pour la détection de *S. haematobium*, une analyse quantitative de l'urine sera réalisée en utilisant la technique de filtration. Tout échantillon dépisté positif pour l’infection à *S. haematobium* sera réexaminé le lendemain, si bien que pour chaque malade, il y aura deux filtres. Le contrôle de qualité des résultats portera sur la relecture de 10% de l’ensemble des filtres choisis au hasard par un lecteur expérimenté d’une autre unité du DEAP ou d’un autre laboratoire. Si la différence (différence de 10 œufs) entre le nombre d’œufs comptés par les deux lecteurs est inférieure à 20% pour tous les filtres lus (exceptés pour les cas des enfants hyperparasités), la moyenne de la parasitémie sera calculée et utilisée pour l’analyse. Si la différence entre les deux lecteurs est égale ou supérieure à 20% des cas, un troisième lecteur devra relire tous les filtres.

Les enfants respectant tous les critères d'inclusion et qui ne serait frappé d’aucun critère de non inclusion auront un numéro d’étude correspondant à un des bras de traitement alloué de façon aléatoire.

Les enfants infectés qui ne seront pas inclus dans l'étude seront traités selon les directives actuelles de traitement de la bilharziose du pays.

**4.9.2 Traitements**

**4.9.2.1. Médicaments**

Pendant les 2 premiers jours de l'étude (jours 0-1), les malades inclus seront admis pour la prise du médicament et l’observation. Tous les traitements seront donnés au centre de santé par un personnel qualifié, et le dosage se fera en fonction du poids. Tous les traitements seront administrés par voie orale. Les sujets seront soumis à un des bras de traitement de façon aléatoire double aveugle et tous les malades recevront leurs médicaments en deux jours. Au jour 0 le groupe PZQ recevra sa dose (40 mg/kg, comprimés de 400mg, dose orale en une seule prise). Mais pour respecter le double aveugle de l'essai, les malades assignés au groupe As+SMP recevront des placebos ce jour 0 (le nombre de comprimés sera aussi en fonction du poids). Tous les comprimés non administrés seront détruits. Le jour suivant les malades ayant reçu le PZQ le jour précédent recevront 3 comprimés de placebo en 24 heures. Les malades qui avaient reçu des comprimés de placebo le jour 0 recevront 3 comprimés de As+SMP en 24 heures. Au cas où le malade vomirait dans les 30 minutes qui suivent l'administration de la dose, une seconde dose complète lui sera administrée. En cas de vomissement 30 à 60 minutes après administration, une demi dose lui sera administrée à nouveau. Si le malade rejette encore ses comprimés, il recevra un autre médicament et sera retiré de l'étude (arrêt prématuré). Un mode opératoire normalisé pour le traitement (selon le fabriquant des médicaments) sera disponible pour le personnel médical pour une administration appropriée des doses des différents médicaments. Le nombre de comprimés administrés les jours de traitement et en cas de vomissement doit être enregistré dans le CRF.

10% double contrôle

Evaluation Inclusion dans l’essai Analyse finale

Préliminaire (Jour -1) Résultats reportés

↓ Un mois d’intervalle, pas de traitement

↑ ↑ ↑ ↑ ↑ ↑

Dépistage Traitement Final évaluation

2 échantillons d’urine Jour 0: PZQ, Pb 2 échantillon d’urine

(Jour-3, -2) Jour 1: Pb, As+SMP (Jour 28, 29)

**4.9.2.2. Médicaments concomitants**

Les enfants peuvent recevoir parallèlement d’autres médicaments à la discrétion du clinicien de l’étude. Mais, toute utilisation concomitante de médicaments (y compris le paracétamol et les médicaments contre le HIV) sera documentée dans le formulaire de report des cas (FRC).Toutefois, l’utilisation d’autres antibilharziens (tel que l’oxaminiquine ou le métriphonate) et les ACT contre l’infection palustre ne sera pas admise, car ces médicaments pourraient perturber l’interprétation correcte de la réponse aux médicaments de l'étude.

**4.9.3 Poursuite du traitement**

Après inclusion dans l'essai, les malades seront invités à revenir pour une évaluation 28 jours après l’administration des médicaments. Au cours de la période précédant les évaluations (mois de l'étude), les enfants tireront profit de la présence permanente des cliniciens au centre de santé. Cette présence permettra aussi aux cliniciens d’assurer le suivi des enfants pour l’apparition des éventuels signes cliniques, des événements adverses et leur évolution. Les effets adverses seront en particulier suivis avec la plus grande attention au cours des 3 premiers jours qui suivront l’administration du médicament. Après les 3 jours initiaux, les cliniciens resteront néanmoins au centre de santé pour la prise en charge des événements adverses tardifs. L'événement adverse sera défini comme tout changement défavorable ou involontaire sur le plan anatomique, physiologique ou métabolique se rapportant à un signe physique, un symptôme et/ou des changements détectés par le laboratoire qui se produit au cours de l'étude considérée ou non et pouvant avoir un lien avec l'étude. Cela inclut une aggravation des conditions préexistantes et les cas de maladie intercurrente. Les événements adverses sérieux seront définis par:

- - La menace de mort: elle est définie comme un évènement qui expose à un risque immédiat de mort et ne s’applique pas à un évènement qui de façon hypothétique pourrait causer la mort s’il était plus sévère;
  - L’hospitalisation au cours de l’étude: elle est définie comme un séjour d’au moins une journée à l’hôpital ou en salle d’urgence pour des soins qui ne pourraient être administrés en ambulatoire;
  - Invalidité ou incapacité : définie comme étant une perturbation importante des facultés du participant l’empêchant de mener des activités ordinaires ;
  - Tous les autres problèmes médicaux qui ne peuvent pas conduire à la mort, tels que la menace de mort ou une hospitalisation recommandée seront considérés comme évènements indésirables si la vie du sujet est en danger nécessitant une intervention chirurgicale ou médicale pour prévenir la survenue de l’un de ces évènements ;
  - La grossesse
  - Le décès au cours de l’étude.

Les évènements indésirables biologiques seront définis sur la base des tableaux suivants:

**Tableau I**: Intervalles de référence** des valeurs Hématologiques chez les enfants de 6-14* ans

| **Paramètres** | **Unités** | **Intervalle de référence** | **90% Intervalle de Confiance de la limite inférieure** | **90% Intervalle de confiance de limite supérieure** |
| --- | --- | --- | --- | --- |
| **WBC** | 10^3^/μL | 4.5 – 10.5 | 4.2 – 4.9 | 9.9 – 12.0 |
| **RBC** | 10^6^/μL | 3.58 – 5.17 | 3.27 – 3.68 | 5.02 – 5.34 |
| **Hémoglobine** | g/dL | 9.6 – 13.5 | 9.3 – 9.8 | 13.1 – 14.2 |
| **Hématocrite** | % | 29.3 – 41.3 | 26.9 – 30.4 | 39.9 – 42.5 |
| **MCV** | fL | 70.5 – 94.2 | 64.3 – 72.1 | 89.2 – 95.1 |
| **MCH** | pg | 21.8 – 30.5 | 21.2 – 22.7 | 29.4 – 32.2 |
| **MHCH** | g/dL | 30.5 – 34.5 | 30.0 – 30.8 | 34.2 – 35.3 |
| **Nombre de Plaquettes** | 10^3^/μL | 138– 455 | 91 – 167 | 436 – 533 |

** Basé sur le classement des enfants en tranche d’âge de 6 à 14 ans.*

*** Basé sur l’échnatillonnage realisé en octobre*

**Tableau II :** Intervalles de référence** des valeurs de biochimie chez les enfants de 6-14* ans

| **Paramètres** | **Unités** | **Intervalle de réféerence** | **90% Intervalle de Confiance de la limite inférieure** | **90% Intervalle de Confiance de la limite supérieure** |
| --- | --- | --- | --- | --- |
| **ALT***** | U/L | 5.06 – 53.4 | 3.10 – 6.99 | 34.60 – 74.50 |
| **Créatinine** | μM/L | < 48.9 | N/A | 46.8 – 49.6 |

** Basé sur le classement des enfants en tranche d’âge de 6 à 14 ans.*

*** Basé sur l’échnatillonnage realisé en octobre*

*** à 37^o^C

Les symptômes seront liés à l’administration du médicament si les mêmes symptômes n'avaient pas été rapportés à la première consultation clinique durant le dépistage et que le clinicien n'ait pas trouvé une autre cause à sa survenue.

Tous les évènements adverses doivent être rapportés sur la FCR. Les évènements adverses qui sont sérieux ou inattendus seront communiqués par fax à Dafra Pharma nv dans les 24 heures qui suivent leur apparition. Dafra Pharma nv informera à son tour le Comité d’éthique par le biais de l'investigateur principal dès que possible. La fiche des événements adverses sérieux sera remplie et attachée au CRF.

Un mois plus tard, une nouvelle évaluation des paramètres (interrogatoire, examen physique et clinique, évaluation parasitologique, évaluation de l'hématurie, évaluation hématologique, évaluation biochimique) sera conduite et les informations seront enregistrées dans le CRF.

Pour l'évaluation parasitologique, 2 échantillons d’urine de chaque malade seront collectés pendant 2 jours consécutifs et analysés afin d’augmenter la sensibilité et réduire les effets de variation quotidienne dans le comptage des œufs.

Les malades qui ne sont pas présents le jour du rendez-vous seront contactés pour le suivi. Ils seront cherchés systématiquement par les guides locaux. Les malades perdus de vue seront retirés de l'essai et ne seront pas inclus dans l’analyse.

**4.9.4 Examens de laboratoire**

Tous les tests de laboratoire seront effectués par le laboratoire clinique du MRTC utilisant les techniques standards.

- **Recherche parasitologique**

Pour détecter les œufs de *S. haematobium*, un échantillon de 10 ml d'urine sera prélevé (de préférence au milieu de la miction) entre 10 heures et 14 heures pendant 2 jours consécutifs. Un échantillon d'urine pris au milieu de la miction est un échantillon dans lequel la première moitié de l’urine est versée alors que la seconde moitié est retenue pour le prélèvement.

Après avoir noté la présence ou non de sang dans les échantillons d’urine (voir ci-dessous la détection de l'hématurie), les urines seront analysées pour le diagnostic de *S. haematobium* en ajoutant 5 ml de colorant (1% de carbol-fuchsin) à 10 ml de l'échantillon. Ce mélange passe à travers un système de filtration utilisant une membrane microscopique. Les œufs sont retenus sur la membrane, puis colorés à l’aide d’une goutte de solution de Lugol et comptés sous microscope (5). Le contrôle de la variabilité du nombre d’œufs entre les lecteurs se fera par la lecture de 10% des membranes par un second microscopiste expérimenté indépendant.

Les œufs seront quantifiés avant et après l'étude comme suit:

- négatif : absence d’œufs

- Faibles excréteurs : 1-49 œufs/filtre

- Forts excréteurs : ≥50 œufs/filtre

La charge parasitaire peut être déduite de ces informations. La femelle *S. haematobium* produit entre 20 et 300 œufs par jour (4). La moyenne géométrique (Le nombre d’œufs +1) sera utilisée pour mesurer le nombre d’œufs. La réduction du nombre d’œufs sera calculée comme (1-(MG du nombre d’œufs après traitement/MG du nombre d’œufs avant traitement)) X 100 (10).

### Détection de l’hématurie

Avant de mélanger l'échantillon d'urine avec la solution de colorant l'aspect macroscopique de chaque échantillon d'urine doit être noté (clair, trouble ou taché de sang). L’hématurie sera détectée dans les échantillons en utilisant les bandelettes réactives (Hematix® Bayer). Les résultats seront enregistrés comme suit (5):

- négatif (aucune réaction)

- trace (sang non hémolysé)

- + (environ 25 érythrocytes/µl)

- ++ (environ 80 érythrocytes/µl)

- +++ (environ 200 érythrocytes/µl)

- **Hématologie et Biochimie**

Les données suivantes seront collectées pour évaluer la tolérance clinique et biologique:

- - Hémogramme: GB (Globules blancs), GR (Globules rouges), Hémoglobine, Hématocrite, VGM (Volume Globulaire Moyen), TGMH (Teneur Globulaire Moyenne en Hémoglobine), CGMH (Concentration Globulaire Moyen en Hémoglobine) et Plaquettes.
  - Bilan hépatique: ALAT
  - Bilan rénale: créatinine
  - Test de grossesse : Ce test sera réalisé pour les volontaires de sexe féminin ayant 11 ans et plus au moment du dépistage après un conceling avec les parents ou le conjoint.

**4.9.5 Plan de supervision**

La supervision interne sera faite systématiquement par l'Investigateur Principal pendant l'inclusion et la période d’évaluation. La supervision externe sera faite par Dafra Phama nv au début et à la fin de l'étude.

Le site de l’étude sera visité par deux membres du comité d’éthique de la faculté de médecine, de pharmacie et d’odontostomatologie durant la période de suivi des malades.

**4.10 CONSIDERATIONS ETHIQUES ET DEONTOLOGIQUES**

Le protocole de recherche sera soumis à l'approbation du Comité d'Ethique Institutionnel. Tout amendement subséquent fera l’objet d’une soumission au même comité. Sur le site, la communauté (autorités administratives, règles coutumières, malades ou personnes en charge des jeunes malades) sera informée de tous les aspects de l'étude. La participation à l'étude sera entièrement volontaire. Aucun sujet ne sera inclus contre son gré.

Chaque sujet adulte signera ou apposera son empreinte digitale sur la fiche de consentement volontaire qui lui sera présentée par l’équipe de recherche. Pour les mineurs, les parents ou tuteurs signeront ou apposeront leur empreinte digitale. Pour les sujets qui ne lisent pas le français, le consentement éclairé sera traduit dans la langue locale en présence d'un témoin lettré de la communauté.

**4.10.1 Plan de soins des malades**

***Echantillons d'urine.*** Le protocole sera strictement appliqué par le personnel médical et paramédical qualifié. L'équipe de recherche respectera les directives de bonnes pratiques cliniques et de laboratoire. Pour prévenir toute contamination causée par des instruments souillés avec l’urine, les selles, le sang ou autres liquides corporels, chaque examen sera réalisé en utilisant des instruments stériles à usage unique. Des antiseptiques seront utilisés pour désinfecter les régions contaminées (corporels ou autres) ou les régions qui peuvent être contaminées. En raison du risque élevé de l'infection HIV de nos jours, des mesures de protection personnelle strictes seront prises ainsi que la désinfection de toutes les surfaces et régions de contact du corps.

***Arrêt du traitement/ou Institution d’un traitement de recours.***

Tous les malades impliqués dans l'étude qui souffriraient de maladies contractées au cours de l'étude recevront un traitement complet et gratuit selon les standards locaux.

Si les malades rejettent les médicaments de l'étude (vomissements à plusieurs reprises dans les 60 minutes de suivi), les sujets seront retirés de l'étude mais leurs données seront prises en compte dans l’analyse respective du bras de traitement. Le malade sera considéré comme un cas d’échec (non guéri) pour le résultat primaire et les parents/tuteurs seront informés de la raison de son retrait de l’étude. Le malade sera traité pour bilharziose en utilisant un autre médicament disponible.

Toutes les raisons médicales qui justifieraient le retrait d’un malade de l’étude seront clairement expliquées au malade ou au parent/tuteur.

**4.10.2 Confidentialité des résultats**

Les dossiers des malades seront confidentiels et ne seront accessibles qu’après l’approbation de l'investigateur principal selon les principes d’éthique et de protection des sujets. Les échantillons ne porteront pas le nom du malade. Les malades ne seront pas identifiés dans aucun rapport ou publication.

**4.10.3 Risques potentiels et effets indésirables**

Les informations détaillées sur les effets adverses possibles des médicaments de cette étude aussi bien que leurs contre-indications seront fournies en annexe.

**4.10.4 Risque et minimisation du risque**

Les risques encourus sont minimes. Le prélèvement de sang pourrait causer des désagréments et des malaises au point de prélèvement. Ces tests sont non invasifs et nous n’en connaissons aucun évènement adverse. La prise de sang peut causer des malaises et des douleurs occasionnelles au point de prélèvement, et rarement, l'évanouissement. Nous nettoierons le bras ou le doigt de votre enfant avant tout prélèvement de sang et utiliserons de nouvelles aiguilles pour prélever le sang. Chez de nombreux enfants la prise des médicaments ne pose aucun problème, mais souvent la prise du Co-Arinate FDC, les enfants pourraient présenter de légères éruptions ou des maux de ventre. Quelques effets secondaires tels les maux de tête, les douleurs abdominales et les nausées pourraient également accompagner la prise du praziquantel quand cette prise se faisait chez le sujet à jeun. Les médicaments utilisés pour traiter le paludisme et les vers sont ceux déjà utilisés par le Programme National de Lutte Contre les Schistosomoses et le Programme de Lutte Antipaludique dans notre pays.

Les sujets bénéficient d'un suivi adéquat sur le plan clinique et biologique. En raison de la présence constante des médecins de l'équipe de recherche dans la communauté pendant la période de l’étude, les cas de résistance et d’échec du traitement sont diagnostiqués et pris en charge précocement.

Chaque fois qu’un participant ne se sentira pas bien après la prise des médicaments, il lui sera recommandé de se rendre au centre de santé. Si le participant est un enfant, le parent/personne en charge sera invité à conduire l'enfant au centre de santé. Les critères objectifs pour l’arrêt prématuré du traitement et la mise en route d’un traitement de recours ou l’incapacité de prendre le médicament seront considérés comme des effets adverses sérieux.

**4.10.5 Risques, bénéfices et compensation**

Pour les malades inclus dans l'étude, les coûts d'évacuation et d’hospitalisation pour les cas sévères seront à la charge du projet. Pour tous les malades inclus dans l'étude un suivi médical rapproché et gratuit leur sera accordé. Le rapport risques/bénéfices de cette étude favoriserait la participation des malades.

Tous les participants seront informés qu’aucun malade inclus dans cet essai ne recevra directement de l'argent pour sa participation à l'étude.

**4.10.6 Publication des résultats**

A la fin de l'étude, la population de Djalakorodji, le Programme National de lutte contre la Bilharziose et le promoteur Dafra Pharma nv seront informés des résultats obtenus. Les résultats seront également présentés lors de rencontres nationales et internationales et seront publiés dans des journaux scientifiques. Cependant, l’équipe de recherche garantit le caractère confidentiel des informations individuelles collectées sur les malades. Ces informations ne seront accessibles qu’aux investigateurs impliqués dans cette étude.

**5. Références**

1. Utzinger, J., Keiser, J., Shuhua, X., Tanner, M., Singer B.H.. 2003 **Combination chemotherapy of schistosomiasis in laboratory studies and clinical trials**. *Antimicrob. Agents Chemother*, May; pp 1487-1495.
2. N’Goran, E.K., Utzinger, J., Gnaka, H., Yapi, A., N’Guessan, N.A., Kigbafori, S.D., Lengeler, C., Chollet, J., Shuhua, X., Tanner, M.; 2003 **Randomized, double-blind, placebo-controlled trial of oral artemether for the prevention of patent *Schistosoma haematobium* infections**. *Am. J. Trop. Med. Hyg*., 68(1), , pp 24-32.
3. Markell, E., John, D., Krotoski, W. 1999 **‘Markell and Voge’s Medical Parasitology**.’ 8^th^ edition, W.B. Saunders Company, Philadelphia.
4. Utzinger, J., Xiao, S.H., Tanner, M., Keiser, J. 2007 **Artemisinins for schistosomiasis and beyond**. *Curr. Opin. Investig. Drugs*. Feb; 8(2): pp 105-116.
5. Inyang-Etoh, P.C., Ejezie, G.C., Useh, M.F., Inyang-Etoh, E.C. 2004 **Efficacy of artesunate in the treatment of urinary schistosomiasis in an endemic community in Nigeria.** *Ann. Trop. Med. Par*.; 98(5), pp. 491-499.
6. Fenwick, A., Keiser, J., Utzinger, J. 2006 **Epidemiology, burden and control of schistosomiasis with particular consideration to past and current treatment trends.** *Drugs Fut*; 31(5), pp 413-425.
7. Cheesbrough, M. 1998 ‘**District Laboratory Practice in tropical countries, Part 1.**’ Cambridge University Press, Cambridge.
8. **Mali- First year of baseline data collection completed (March-April 2004).,** Schistosomiasis Control Initiative 2007, Imperial College London, London. Viewed 23/04/2007, <http://www.schisto.org/Mali/1stBaseline.htm>
9. **Methods in parasitology: Kato-Katz technique for helminth eggs.** Swiss Tropical Institute, Basel. Viewed 24/04/2007, <http://www.tropeduweb.ch/Parasitology_Methods_PDF/8_Stool_Kato-Katz.pdf>
10. De Clercq, D., Vercruysse, J., Verlé, P., Kongs, A., Diop, M**.** 2000 **What is the effect of combining artesunate and praziquantel in the treatment of *Schistosoma mansoni* infections?** *Trop. Med. Int. Health;* 5(10), pp 744-746.
11. Borrmann, S., Szlezak, N., Faucher, J.F., Matsiegui, P.B., Neubauer, R., Binder, R.K., Lell, B., Kremsner, P.G. 2001 **Artesunate and praziquantel for the treatment of Schistosoma haematobium infections: a double-blind, randomized, placebo-controlled study*.***  *J. Infect. Dis*. Nov 15; 184(10):pp 1363-6.
12. De Clercq, D., Vercruysse, J., Kongs, A., Verle, P., Dompnier, J.P., Faye, P.C. 2002 **Efficacy of artesunate and praziquantel in Schistosoma haematobium infected schoolchildren.** *Acta Trop.* Apr; 82(1):pp 61-6.
13. Adam, I., Elhardello, O.A., Elhadi, M.O., Abdalla, E., Elmardi, K.A., Jansen, F.H. 2007 **Antischistosomal efficacy of artesunate plus sulfamethoxypyrazine/pyrimethamine and artemether-lumefantrine administered as treatment for uncomplicated *Plasmodium falciparum* malaria.**

*In Press.*

1. Sissoko K. **Impact de l’infection à Schistosoma haematobium sur les paramètres paludométriques dans un village d’endémie palustre au Mali.** *Thèse Médecine, 2005, 47p.*
2. Doumbia S. **Evolution des paramètres paludométriques au cours de la coinfection Schistosoma haematobium et Plasmodium falciparum dans un village au Mali.** *Thèse Médecine, 2007, 85p.*

**ANNEXE A**

**Consentement éclairé pour parent ou tuteur**

**Titre du Projet:** Essai clinique randomisé double aveugle comparant l’efficacité de l’artesunate + sulfamethoxypyrazine/pyrimethamine versus praziquantel dans le traitement de la bilharziose urinaire chez les enfants.

Numéro de dépistage du Patient …………………… Numéro d’étude du Patient …………………

Nom du Parent/Tuteur ……………………………………

**1. But**

Nous invitons votre enfant à prendre part à une étude portant sur la comparaison de l’efficacité de deux médicaments contre la schistosomose : le praziquantel déjà utilisé par le programme et le Co-Arinate FDC® utilisé contre le paludisme. Cette étude devra s’effectuer chez tous les enfants de 7-15 ans excrétant des œufs de schistosome dans leurs urines. Nous voulons savoir quel est le médicament le plus efficace dans le traitement de cette maladie. Le paludisme est une maladie causée par un très petit parasite qui peut entrer dans le corps quand un moustique infecté vous pique. La maladie peut entraîner des maux de tête, de la fièvre, des vomissements et beaucoup d’autres malaises. La bilharziose est une maladie qui se contracte par le contact de la peau avec l’eau contaminée en s’y baignant ou en y effectuant certaines activités comme la culture irriguée, la pêche, la lessive ou le lavage des ustensiles de cuisine etc. Toutefois, toutes ces maladies peuvent être traitées et complètement guéries, si elles sont diagnostiquées précocement. Le but de ce travail est d’évaluer l’efficacité du praziquantel et du Co-Arinate FDC® dans le traitement de la bilharziose. Notre étude tente de montrer qu’il existe de nouveaux médicaments pouvant bien traiter la bilharziose. L’intérêt majeur du médicament que nous proposons est qu’il est aussi efficace contre le paludisme, pour le malade. Un total de 800 malades sera inclus dans cet essai et tous ces malades seront étudiés au centre de santé de la localité.

**2. Procédure**

La participation de votre enfant consistera à lui faire subir un interrogatoire, un examen physique (pour savoir s’il n’a pas une autre maladie), des analyses de sang, de selles et d’urines. Chaque participant recevra une piqûre au doigt pour l’équivalent de 3 ml de sang (l’équivalent de moins d’une cuillerée à café). Nous procéderons au nettoyage des doigts avec de l’alcool et le prélèvement se fera toujours avec des instruments stérilisés et à usage unique. Une analyse de ce sang sera faite pour déterminer l’effet du traitement sur la composition du sang. Il y aura donc deux séries de prélèvement, l’une avant l’étude et l’autre à la fin de l’étude. La première permettra de savoir quelle est la composition exacte du sang à J0 et la seconde à J28 permettra de savoir quelles sont les changements induits par le traitement en comparant la composition du sang avant et après traitement. L’examen des urines permettra de savoir si les urines contiennent ou non des œufs de bilharzie. Les urines sont recueillies dans des sachets plastiques sur lesquels nous aurons inscrit le numéro de votre enfant. Les urines seront collectées entre 10H et 14H heures favorables à une production maximale des œufs chez les schistosomes. Une fois les sachets distribués, les enfants pourront revenir avec les urines et les déposer dans une bassine dont l’emplacement leur sera indiqué. Aussitôt après usage, le reste des urines et les sachets sont détruits sur place. Un test de grossesse sera effectué sur les filles âgées de 11 – 15 ans pour éviter l’absorption de ces médicaments par de telles filles.

Come vous le constatez le temps de suivi après traitement est de 28 jours, la participation de votre enfant à cette étude sera donc conditionnée à sa présence effective dans le village pendant toute la durée de l’étude. Dès la fin des examens de sang et d’urine, nous procéderons au traitement de votre enfant soit par le praziquantel soit par la Co - Arinate FDC®. Mais ni moi ni le médecin traitant ne saurons quel médicament il a reçu. Ce sera à la fin de l’étude que le secret sera dévoilé. Votre enfant recevra les deux médicaments au centre de santé au bout de deux jours.

**3. Participation**

La participation de votre enfant est volontaire et vous pouvez mettre fin à cette participation à n'importe quel moment sans qu’il ne perde les avantages liés à celle-ci. Si après traitement de votre enfant les parasites persistent, il recevra d’autres soins jusqu’à la guérison. Nous vous inviterons à revenir après 28 jours et nous vérifierons encore les échantillons d’urine de votre enfant pour les parasites. Si nous trouvons encore des parasites, votre enfant continuera à recevoir le traitement jusqu'à la disparition des parasites. Si votre enfant commence à développer une maladie quel que soit le symptôme ou le signe avant le 28^ème^ jour, vous devriez le conduite aussitôt au centre de santé ou une équipe sera installée de façon permanente pendant toute la durée de l’étude. La durée entière de la participation dans cet essai sera d’un mois (Jours de visites -3, -2, 0,1, 28 et 29).

**4. Risques et Bénéfices**

Les risques encourus sont minimes. Le prélèvement de sang pourrait causer des désagréments et des malaises au point de prélèvement. Ces tests sont non invasifs et nous n’en connaissons aucun évènement adverse. La prise de sang peut causer des malaises et des douleurs occasionnelles au point de prélèvement, et rarement, l'évanouissement. Nous nettoierons le bras ou le doigt de votre enfant avant tout prélèvement de sang et utiliserons de nouvelles aiguilles pour prélever le sang. Chez de nombreux enfants la prise des médicaments ne pose aucun problème, mais souvent la prise du Co-Arinate FDC, les enfants pourraient présenter de légères éruptions ou des maux de ventre. Quelques effets secondaires tels les maux de tête, les douleurs abdominales et les nausées pourraient également accompagner la prise du praziquantel quand cette prise se faisait chez le sujet à jeun. Les médicaments utilisés pour traiter le paludisme et les vers sont ceux déjà utilisés par le Programme National de Lutte Contre les Schistosomoses et le Programme de Lutte Antipaludique dans notre pays.

La participation de votre enfant sera bénéfique pour la communauté en aidant les médecins à choisir le médicament le plus efficace entre les deux proposés et comment. Tous les tests qu’auront subis les enfants de même que les médicaments qui leur seront donnés seront gratuits. Tous les problèmes potentiels relatifs au traitement seront pris en charge gratuitement par les médecins de notre équipe et notre partenaire Dafra Pharma nv..

**5. Confidentialité**

Nous prenons l’engagement que les résultats de ces tests seront confidentiels. Une copie de ce formulaire de consentement contresigné sera placée dans votre dossier au niveau du laboratoire. Les résultats pourraient être publiés dans des revues médicales ou dans des conférences mais votre nom (ou celui de votre enfant) ne sera en aucun cas utilisé dans les rapports. Les informations spécifiques recueillies sur votre enfant ne seront en aucun cas partagées avec d’autres personnes exceptées les investigateurs de l’étude.

**6. Droit au retrait**

La participation à cette étude est entièrement volontaire. Vous pouvez refuser que votre enfant y participe ou le retirer de l’étude à tout moment sans aucune sanction ni la perte du droit aux soins requis en cas de diagnostic d’une maladie parasitaire. L’investigateur en charge de ce travail de recherche a aussi le droit de mettre fin à cette participation à tout moment si une raison médicale l’exigeait et dont la nature vous sera précisée, ou pour tout autre motif incompatible avec le bon déroulement de l’étude.

## 7. Coût et Compensation

Tous les soins médicaux incluant les contrôles de santé, les analyses de sang, de selles et d’urines seront faits gratuitement. Que vous décidiez de participer à cette étude ou non, nous procéderons au traitement des infections parasitaires que nous diagnostiquerons dans le sang, les selles et les urines. Des soins adéquats seront administrés à chaque volontaire indépendamment de son statut à l’étude et selon les bonnes pratiques cliniques. Les compensations en nature ou en espèce ne sont toutefois pas prévues pour la participation de votre enfant à cette étude en dehors du traitement reçu.

## 8. Consentement

Je confirme avoir reçu toutes les informations concernant la participation de mon enfant à cette étude par Mr …………………………………… J’ai compris ce qu’on demande et ce que subira mon enfant en participant à l’étude. J’ai également compris que je pourrais décider de retirer mon enfant au cas où je le jugerai opportun sans risque de perdre les avantages liés à cette étude. Si vous avez des questions ou des inquiétudes pour la participation de votre enfant à cette étude, vous pourrez en parler avec un des membres de notre équipe ou demander à votre chef de quartier d’envoyer une lettre au Dr. Mahamadou Soumana Sissoko à la Faculté de Médecine de Pharmacie et d’Odonto-Stomatologie du Point-G à Bamako (BP. 1805 ; Tel/Fax: (223) 222 81 09) ou au secrétariat permanent du comité d’éthique à la FMPOS (Tél : (223) 222 52 77 Poste 113). Les médecins du centre de Santé vous aideront aussi à contacter les personnes ressources.

_______________ ______________________ __________

Empreinte digitale ou Signature du Tuteur date

______________________ __________

Signature de l’investigateur date

______________________ __________

Signature du témoin date

**ANNEXE B**

**BUDGET**

| **Items** | **Budget en Euro** |
| --- | --- |
| Personnel  Equipement et Réactifs  Frais opérationnels (Logistique, consommables, communication, soins médicaux, compensation) | 52.800  54.400  36.800 |
| Frais institutionnels (10% du budget) | 16.000 |
| **Total** | **160.000** |

**Pourcentage d’effort du personnel clef :**

Prof. Ogobara Doumbo : 8% : Rôle dans la conception de l’étude, la direction scientifique et le maintien du contact avec les autorités sanitaires et universitaires, la prise en charge et la gestion des évènements indésirables (EIs), l’analyse et l’interprétation des données.

Dr Mahamadou Soumana Sissoko : 25% : Chercheur principal, rôle dans la conception de l’étude, la planification opérationnelle, la conduite et la supervision de l’étude, la prise en charge et la gestion des EIs, l’obtention de la permission communautaire et l’administration du consentement éclairé, l’analyse et l’interprétation des données, la rédaction des différents rapports relatifs à l’étude.

Prof Abdoulaye Dabo : 25% : Rôle dans la conception de l’étude, l’obtention de la permission communautaire et l’administration du consentement éclairé, le contrôle de qualité interne des données, l’analyse et l’interprétation des données, la rédaction des différents rapports relatifs à l’étude et responsable des examens d’urine.

Dr Saïbou Doumbia : 100% Rôle dans la conception de l’étude, la coordination clinique de l’étude, le monitoring interne de l’étude, l’obtention du consentement éclairé, la prise en charge et la gestion des EIs, le contrôle de qualité interne des données.

Dr Niawanlou Dara : 100% : Rôle dans la conception de l’étude, pharmacien de l’étude en charge des produits d’investigation et technicien pour les examens d’urine.

Mr Ousmane Touré : 25% : Rôle dans la conception de l’étude, la gestion des données et l’analyse statistique de l’étude, l’élaboration du rapport final.

**ANNEXE C : CURRICULUM VITAE DES CHERCHEURS**
